# Do children’s upper respiratory tract infections benefit from probiotics?

**DOI:** 10.1186/1471-2334-14-194

**Published:** 2014-04-10

**Authors:** Susanna Esposito, Donato Rigante, Nicola Principi

**Affiliations:** 1 Pediatric Highly Intensive Care Unit, Department of Pathophysiology and Transplantation, Università degli Studi di Milano, Fondazione IRCCS Ca’ Granda Ospedale Maggiore Policlinico, Via Commenda no. 9, Milan 20122, Italy; 2 Institute of Pediatrics, Università Cattolica Sacro Cuore, Rome, Italy

**Keywords:** Acute otitis media, Children, Prevention, Probiotics, Respiratory tract infection, Upper respiratory tract infections

## Abstract

**Background:**

The microbiota of the gastrointestinal tract have profound influence at multiple levels, even on the development and maintenance of lung immunity and inflammation. Aim of this review is to evaluate the current knowledge about the specific impact on children’s respiratory tract infections from probiotics, live microbes with the power to modify intestinal microbial populations and exert subsequent benefits for the host.

**Discussion:**

The role of probiotics in gastrointestinal and allergic diseases has been largely assessed, but the number of studies performed so far in the field of respiratory tract infections is small, though some data show that probiotic administration might display clinical advantages. Probiotic strain identity and host genetic differences may account for differential modulation of immune responses by probiotics. Current laboratory and clinical data regarding the possibility of the role of probiotics on preventing the development of respiratory tract infections are contradictory, and are somewhat insufficient to recommend strongly their routine use. Further study of gastrointestinal-respiratory interactions is likely to yield important insights into the pathogenesis of different pulmonary diseases, and improve our knowledge in the prophylactic role of probiotics in children affected by recurrent upper respiratory tract infections.

**Summary:**

A better understanding of the effects of different probiotic strains and a deeper insight into their mechanisms of action are needed for the validation of specific strains carrying a potential to modify the frequency and severity of RTIs in infants and children. No data have been collected in pediatric patients with chronic underlying diseases, and yet there are no published data concerning treatment of RTIs with probiotics. The very few studies published so far do not indicate which micro-organism or administration regimen might exert beneficial effects as a prevention tool of RTIs both in healthy children and in those with recurrent RTIs. Further research to establish the role of probiotics in the treatment and prevention of RTIs, including those involving the lower respiratory tract, are required and should also clarify if any susceptible subgroups of respiratory diseases exist, and how these subgroups benefit from supplementation with certain probiotic strains.

## Background

Understanding the microbiome of the airways in normal subjects is an essential step in overturning our way of thinking to respiratory medicine, as changes in this microenvironment could influence the nosography of upper and lower airways infections and even other clinical outcomes, such as complications after lung surgery. The importance of the gastrointestinal tract microbiota in the generation of mucosal immune responses and mucosal tolerance has been largely documented also in allergic patients [1-3], but its interaction with the respiratory pathology is still far to be elucidated.

Oral probiotics are non-pathogenic live microbes that when delivered in sufficient quantity can promote health. The most widely studied probiotics are of 2 genera: *Lactobacillus* and *Bifidobacterium*; non-viable microbes have less immunological activity and can be rarely associated with adverse effects, such as gastrointestinal symptoms or diarrhea [4]. A range of studies have advocated a role for probiotics in the prevention and treatment of a wide range of disorders, and different micro-organisms administered by means of a nasal or oral spray that are classic airway commensals have been recently included in the probiotic family, with the aim of preventing ear, nose and throat diseases. Albeit with large differences from one micro-organism to another, it is now established that probiotics can produce antimicrobial products capable of eliminating bacterial pathogens [5], blocking toxin-mediated responses [6], interfering with bacteria that are pathogenic for nutrients and adhesion sites and limiting their presence and virulence [7], and modulating systemic immune responses by enhancing humoral and cellular immunity [8]. Interestingly, probiotics administration seems to be effective also in the management of food allergy symptoms but has no effect on the prevention of sensitization [9]. Another major potential benefit of probiotics has been suggested in patients with asthma [9]. Consequently, probiotics have been extensively used in the area of infectious and immune-mediated diseases. Most of the data regarding probiotic use in children have been collected in studies for prevention or treatment of gastrointestinal disorders, such as infectious and antibiotic-associated diarrhea, travellers’ diarrhea, necrotizing enterocolitis, and *Helicobacter pylori* infection [10-12], although a number of recent studies have also investigated the relationship with atopic diseases [2,3,9,13].

Comparative evaluations have shown that not all probiotics have the same biological activity, but each has specific peculiarities in terms of mechanism of action and efficacy. Routine use of probiotics as an additive therapy in subjects with gastrointestinal or atopic diseases is very frequent in the everyday clinical practice, but their administration in children with respiratory tract infections (RTIs) has been poorly studied and evidences about this topic are still insufficient [13]. Main goal of this review is to evaluate the actual knowledge on probiotics in pediatric RTIs and verify which critical points if resolved might contribute to spread their use in infants and children with respiratory problems.

## Discussion

### Respiratory tract infections and probiotics: a misunderstood relationship

There are no data regarding the use of ingested probiotics to treat RTIs; the only studies available are limited to prevention and are mainly related to the field of upper respiratory tract infections (URTIs). A recent Cochrane meta-analysis of 10 clinical trials involving a total of 3,451 infants, children and adults, found that probiotics were more beneficial than placebo in terms of infection prevention, and reduced the rate of acute URTI and frequency of antibiotic use, but did not decrease the duration of each single episode [14]. The results of the 7 trials involving only infants and children were likewise similar [15-21]. Table 1 summarises the main characteristics of these studies and their conclusions, suggesting that probiotic administration could be a valuable approach to preventing URTIs in children, mostly those with a history of very frequent recurrences. One of the most interesting study has been published by Hojsak *et al*. who showed that children treated with *Lactobacillus rhamonosus* GG compared with those who received the placebo had a significantly reduced risk for gastrointestinal infections (relative risk [RR]: 0.40 [95% confidence interval (CI): 0.25-0.70]; number needed to treat: 15 [95% CI: 9-34]), respiratory tract infections (RR: 0.38 [95% CI: 0.18-0.85]; number needed to treat: 30 [95% CI: 16-159]), vomiting episodes (RR: 0.5 [95% CI: 0.3-0.9]), diarrheal episodes (RR: 0.24 [95% CI: 0.10-0.50]), episodes of gastrointestinal infections that lasted >2 days (RR: 0.40 [95% CI: 0.25-0.70]), and episodes of respiratory tract infections that lasted >3 days (RR: 0.4 [95% CI: 0.2-0.9]); groups did not differ in hospitalization duration [17]. Moreover, Rautava *et al*. compared infant formula supplemented with the probiotics *Lactobacillus rhamnosus* GG and *Bifidobacterium lactis* BB-12 or placebo administered daily until the age of 12 months and showed that during the first 7 months of life 7 out of 32 (22%) infants receiving probiotics and 20 out of 40 (50%) infants receiving placebo experienced acute otitis media (AOM; RR 0.44 [95% CI: 0.21-0.90]; p = 0.014) and antibiotics were prescribed for 10 out of 32 (31%) infants receiving probiotics and 24 out of 40 (60%) infants receiving placebo (RR 0.52 [95% CI: 0.29-0.92]; p = 0.015) [20]. During the first year of life, 9 out of 32 (28%) infants receiving probiotics and 22 out of 40 (55%) infants receiving placebo encountered recurrent respiratory infections (RR 0.51 [95% CI: 0.27-0.95]; p = 0.022) [21]. These data suggested that probiotics may offer a safe means of reducing the risk of early AOM and antibiotic use and the risk of recurrent respiratory infections during the first year of life. However, many experts have criticized these observations [22], as the meta-analysis included trials with different inclusion and exclusion criteria, different primary and secondary outcomes, different diagnostic criteria, and different follow-up periods. Furthermore, they used different probiotics (5 of the 7 studies used *Lactobacillus rhamnosus* GG alone or in combination with other probiotics, and 2 *Lactobacillus casei* combined with other micro-organisms) at various doses and for different times. Consequently, no definite conclusions could be drawn. In our opinion, although *in vitro* and experimental data suggest that probiotics have some effects, it is not known which is the most effective, which dose and which duration of administration might lead to the best results, or which type of URTI is the most susceptible to probiotic use.

**Table 1:** Clinical trials of probiotics and their use in the prevention of pediatric upper respiratory tract infections (URTIs)

***Author, year of publication, country***	***Age range***	***No. of subjects***	***Types of probiotic***	***Main results***
Caceres *et al*. [15], 2010, Chile	1-5 years	398 (203 T, 195 P)	Milk-based product, *L. rhamnosus* HN001, about 10^8^ CFUs/mL	Non-significant reduction of the number of URTIs per child between groups
Hatakka *et al*. [16], 2007, Finland	10 months-6 years	309 (155 T, 154 P)	Gelatine capsule, a combination of *L. rhamnosus* GG, ATCC 53103, *L. rhamnosus* LC 705, *Bifidobacterium breve* PP, *Propionibacterium freudenreichi* spp. shermanii IS, 8–9 × 10^9^ CFUs of each strain/capsule	Probiotics did not prevent the occurrence of acute otitis media or the nasopharyngeal carriage of otitis pathogens in otitis-prone children; a reduction in the frequency of recurrent respiratory infections was also noted
Hojsak *et al*. [17], 2010, Croatia	13 months-7 years	281 (139 T, 142 P)	Fermented milk product, *L. rhamnosus* strain GG, 10^9^ CFUs	*L. rhamnosus* GG decreased the risk of nosocomial gastro-intestinal and respiratory tract infections in pediatric facilities
Hojsak *et al*. [18], 2010, Croatia	>12 months	742 (376 T, 366 P)	Fermented milk product, *L. rhamnosus* strain GG, 10^9^ CFUs	*L. rhamnosus* GG decreased the risk of upper respiratory tract infections in children attending day care centres
Merenstein *et al*. [19], 2010, USA	3-6 years	638 (314 T, 324 P)	*L. casei* DN-114 001/CNCM I-1518 (also called *L. paracasei* subsp. paracasei in the current nomen-clature), 1 × 10^8^ CFUs/g, *Streptococcus thermophilus* and *L. bulgaricus*, 10 × 7 CFUs/g	Probiotics reduced the overall incidence of common infectious diseases
Rautava *et al*. [20], Finland	0-2 months	81 (38 T, 43 P)	*L. rhamnosus* and *B. lactis* BB-12, 1 × 10^10^ CFUs	Probiotics reduced the risk of early acute otitis media, antibiotic use and recurrent respiratory infections during the first year of life
Rio *et al*. [21], 2009, Argentina	6-24 months	100 (50 T, 50 P)	Fermented milk products, *L. acidophilus* and *L. casei,* 10^7^ to 10^8^/mL	Live *Lactobacillus* supplement suppressed pneumonia and decreased bronchitis in both undernourished and normal subjects

CFUs: colony-forming units; P: placebo; T: treatment.

The role of different probiotics has been evaluated in two works by Weizman *et al*. [23] and Agustina *et al*. [24]. The first was a double-blind placebo-controlled randomised trial of a formula supplemented with *Bifidobacterium lactis* (BB-12), or *Lactobacillus reuteri* 55730, or no probiotics administered for 12 weeks to healthy term-infants aged 4–10 months at 14 child care centres in Israel [23]. In comparison with controls, children treated with BB-12 or *Lactobacillus reuteri* experienced significantly fewer episodes of fever and diarrhea, although there were no between-group differences in the overall incidence of RTIs. The second was a 6-month double-blind placebo-controlled study of 494 healthy children aged 1–6 years, who received low-lactose milk with a low-calcium content (LC 50 mg/day; n = 124), regular calcium content (RC 440 mg/day; n = 126), RC with 5.10^8^ colony-forming units (CFUs) per day of *Lactobacillus casei* CRL431 (n = 120), or RC with 5.10^8^ CFUs per day of *Lactobacillus reuteri* DSM17938 (n = 124): it was found that the incidence of diarrhea episodes was significantly lower in children receiving *Lactobacillus reuteri* DSM17938 than in any other group, whereas *Lactobacillus casei* had no effect. In addition, none of these treatments modified the incidence of RTIs [24].

Different probiotic bacteria have been associated with variable stimuli to the human innate and adaptive immune system and co-mediate metabolic and immune homeostasis, with different levels of success: probiotic strain identity and host genetic differences may account for differential modulation of immune responses by probiotics [25]. Villena *et al*. used an experimental model of lung inflammation based on the administration of the artificial viral pathogen-associated molecular pattern poly(I:C), in order to mimic the pro-inflammatory and physiopathological consequences of RNA viral infections in the lung, and evaluated changes in mouse immunity after oral administration of *Lactobacillus rhamnosus* CRL 1505 and CRL 1506. The authors found that CRL 1506 had no effect, whereas CRL 1505 increased bronchoalveolar lavage concentrations of interleukin(IL)-6, interferon(IFN)-γ and IL-10, and the number of pulmonary CD3 + CD4 + IFN-γ + T cells; the preventive effects on the respiratory airway immunity induced by CRL 1505, suggested that, if the target of probiotic administration is to prevent recurrent URTIs, an appropriate strain of *Lactobacillus rhamnosus* should be selected [26].

Finally, all actual available data show that the combination of different micro-organisms does not always induce more favourable immune modulation, and can lead to negative results on the other hand, underlining the need for specific studies of different probiotic combinations. Hatakka *et al*. performed a 24-week randomized double-blind placebo-controlled interventional study, in which 309 children aged between 10 months and 6 years took one probiotic capsule containing several micro-organisms (*Lactobacillus rhamnosus* GG, ATCC 53103, *Lactobacillus rhamnosus* LC 705, *Bifidobacterium breve* 99 and *Propionibacterium freudenreichii* spp. *shermanii* JS) (n = 155) or placebo (n = 154) once a day [22]: the results of this observation revealed that probiotics did not decrease occurrence, recurrence, or duration of AOM episodes in the population studied (probiotic *vs* placebo respectively: 72% *vs* 65%, *p* > 0.05; 18% *vs* 17%, *p* > 0.05; 5.6 *vs* 6.0 days, *p* > 0.05).

### Administration of probiotics via nasal or oral spray

An alternative to the use of ingested probiotics is to administer airway commensals by means of a nasal or oral spray. This approach is based on the finding that there is a dynamic and antagonistic interaction in the nasopharynx among different colonising organisms, whose manifold infectious potential affects their life cycle, changes the microenvironment, and alters their invasiveness or ability to affect the overall health of the host [27]. Recolonisation with commensal bacteria can reduce the levels of real pathogens and consequently limit the number of new respiratory infections. The first studies were carried out using α *Streptococcus*, showing that a 10-day administration after standard antibiotic therapy reduced the recurrence rates of both pharyngotonsillitis [28] and AOM [29]*.* However, this probiotic approach was subsequently dropped, because of the pathogenicity of α *Streptococcus*, and has only recently been reconsidered using different poorly-infecting bacterial strains capable of producing bacteriocidins.

*In vitro* studies have found that both *Lactobacillus helveticus* MIMLh5 and *Streptococcus salivarius* ST3 can efficaciously adhere to pharyngeal epithelial cells, antagonize *Streptococcus pyogenes*, and modulate host innate immunity, mainly by stimulating the expression of the pro-inflammatory cytokine tumour necrosis factor-alpha [30]. Santagati *et al*. have identified 13 α-hemolytic Streptococci bacteriocidin-producers capable of inhibiting different Gram-positive pathogens, and found that *Streptococcus salivarius* 24SMB does not possess any virulence factor and is a strong producer of bacteriocidins against *Streptococcus pneumoniae*, the most common respiratory bacterial pathogen [31]. However, although these studies provide hopeful indications for the preparation of probiotics in preventing URTIs, human clinical trials have not yet been performed.

### Safety and tolerability concerns for probiotics

Lactobacilli and bifidobacteria have generally been regarded as safe, although there are significant safety concerns in particular populations [32]. A host of experimental data and case reports certify that probiotics might increase the risk of sepsis. Table 2 lists the categories of patients at risk for a probiotic-induced sepsis. The colonisation of neonatal athymic mice with human isolates of *Lactobacillus reuteri, Lactobacillus acidofilus, Bifidobacterium animalis,* or *Lactobacillus rhamnosus* GG can bring about a lethal sepsis [33], suggesting that immune deficiency in the first period of life might increase the risk of probiotic-related sepsis. Furthermore, a number of publications have reported the development of sepsis in adult and pediatric patients receiving probiotic supplements. The most frequently isolated micro-organisms have been *Lactobacillus rhamnosus* GG and *Saccharomyces bulardii*, although their hypothetical greater invasiveness remains to be established. Interestingly, in most of these cases, pulse-field gel electrophoresis of chromosomal DNA restriction fragments showed that the bacteremic and probiotic strains were indistinguishable, confirming a direct relationship between the development of infection and probiotics [34,35]. All cases of probiotic-induced sepsis (including those occurring in children) were diagnosed in immunocompromised patients or subjects with a severe underlying disease. Prematurity can be considered as an additional risk factor for the development of probiotic-induced sepsis, even if probiotic administration has been found effective in reducing the risk of infections in neonates with very low birth weight [36].

**Table 2:** Patients showing high or low risk for a probiotic-induced sepsis

***Type of risk***	***Patients***
High	Immunocompromised patients
	Premature neonates
Low	Patients with a central venous catheter
	Patients receiving probiotics by jejunostomy
	Patients concomitantly receiving broad spectrum antibiotics to which the probiotic is resistant
	Patients receiving probiotics with high mucosal adhesion properties or showing an established pathogenicity
	Patients with cardiac valvular disease (for *Lactobacillus* probiotics only)

The presence in the same patients of a single major or more than one minor risk factor dictates caution when using probiotics.

Another concern associated with use of probiotics is the risk of immune deviation or excessive immune stimulation. Intestinal microbiota play a crucial role in normal immune development, and it cannot be excluded that manipulations designed to alter microbiota by administering probiotics may have significant and persistent immune-modulatory effects. This may be particularly relevant during pregnancy or in the first months of life, because significant abnormalities in microbiota during the first phases of immune system development might lead to major changes in immune responses. It is well known that T cell responses show a bias towards a Th2-phenotype during pregnancy, essential for maintaining fetal viability [37]. As *Lactobacillus* spp. suppresses Th2-cytokine responses *in vitro*, and has been found to increase the production of the Th1-cytokine IFN-γ in some studies, it was initially thought that a probiotic administration might cause fetal loss [38]. Fortunately, there is still no direct evidence of this risk, although further investigation in this field is highly needed [32].

The effect of probiotic administration during pregnancy on infants has been studied in different clinical trials [39-43]. Aim of these studies was to evaluate whether probiotics reduce the risk of developing atopic disease, particularly in children with at least one family member affected by atopic disease. Results were conflicting, and one of the studies clearly showed that probiotics had an unexpected negative effect [40]. This was a study in which 94 mothers received *Lactobacillus* GG (American Type Culture Collection 53103; 5 × 10^9^ CFUs) or placebo twice daily starting 4–6 weeks before expected delivery and continuing after the first 6 post-natal months. Children were monitored for 2 years, and it was found that recurrent (i.e. ≥5) episodes of wheezing bronchitis were more frequent in the *Lactobacillus rhamnosus* GG group (26%; n = 13) than in the control group (9.1%; n = 4), suggesting that the maternal administration of this probiotic may be associated with an increased risk of infectious bronchitis with wheezing in infants for a long time after birth. As no further reports suggesting similar conclusions have been published, it is not possible to establish whether the association was fortuitous or an increased risk of infections exists when the probiotic is given to pregnant women.

## Summary

A better understanding of the effects of different probiotic strains and a deeper insight into their mechanisms of action are needed for the validation of specific strains carrying a potential to modify the frequency and severity of RTIs in infants and children. No data have been collected in pediatric patients with chronic underlying diseases, and yet there are no published data concerning treatment of URTIs with probiotics as well as the possibility to reduce the severity of symptoms. The very few studies published so far do not indicate which micro-organism or administration regimen might exert beneficial effects as a prevention tool of RTIs both in healthy children and in those with recurrent URTIs. Further research to establish the role of probiotics in the treatment and prevention of RTIs, including those involving the lower respiratory tract, are required and should also clarify if any susceptible subgroups of respiratory diseases exist, and how these subgroups benefit from supplementation with certain probiotic strains. Therefore, research activities are focusing currently upon identification of specific probiotic strains with immunomodulatory potential and upon how dietary content interacts with them. Selection of the most beneficial probiotic strain, the dose and timing of supplementation still need to be determined and further study of gastrointestinal-respiratory interactions will yield important insights into the pathogenesis of pulmonary diseases, including cystic fibrosis, respiratory disease of the newborn, and asthma, and improve our knowledge in the prophylactic role of probiotics in children affected by recurrent upper respiratory tract infections.

## Abbreviations

AOM: Acute otitis media; CFUs: Colony-forming units; 95% CI: 95% confidence interval; IL: interleukin; IFN: Interferon; P: Placebo; RR: Relative risk; RTIs: Respiratory tract infections; T: Treatment; URTIs: Upper respiratory tract infections.

## Competing interests

The authors declare no competing interests.

## Authors’ contributions

SE and NP co-wrote the manuscript, revised it for intellectual content, and approved the final version to be published. DR helped draft and revise the manuscript. All authors have read and approved the final manuscript.

## Pre-publication history

The pre-publication history for this paper can be accessed here:

http://www.biomedcentral.com/1471-2334/14/194/prepub
